# Kainate Receptors: Role in Epilepsy

**DOI:** 10.3389/fnmol.2018.00217

**Published:** 2018-06-22

**Authors:** Rafael Falcón-Moya1, Talvinder S. Sihra, Antonio Rodríguez-Moreno

**Affiliations:** ^1^Laboratory of Cellular Neuroscience and Plasticity, Department of Physiology, Anatomy and Cell Biology, University Pablo de Olavide, Sevilla, Spain; ^2^Department of Physiology, Pharmacology and Neuroscience, University College London, London, United Kingdom

**Keywords:** KAR, epilepsy, CA1, MF, dentate gyrus, recurrent mossy fibers, astrocyte

## Abstract

Kainate (KA) is a potent neurotoxin that has been widely used experimentally to induce acute brain seizures and, after repetitive treatments, as a chronic model of temporal lobe epilepsy (TLE), with similar features to those observed in human patients with TLE. However, whether KA activates KA receptors (KARs) as an agonist to mediate the induction of acute seizures and/or the chronic phase of epilepsy, or whether epileptogenic effects of the neurotoxin are indirect and/or mediated by other types of receptors, has yet to be satisfactorily elucidated. Positing a direct involvement of KARs in acute seizures induction, as well as a direct pathophysiological role of KARs in the chronic phase of TLE, recent studies have examined the specific subunit compositions of KARs that might underly epileptogenesis. In the present mini-review, we discuss the use of KA as a convulsant in the experimental models of acute seizures of TLE, and consider the involvement of KARs, their subunit composition and the mode of action in KAR-mediated epilepsy. In acute models, evidence points to epileptogenesis being precipitated by an overall depression of interneuron GABAergic transmission mediated by GluK1 containing KARs. On glutamatergic principal cell in the hippocampus, GluK2-containing KARs regulate post-synaptic excitability and susceptibility to KA-mediated epileptogenesis. In chronic models, a role GluK2-containing KARs in the hippocampal CA3 region provokes limbic seizures. Also observed in the hippocampus, is a ‘reactive plasticity’, where MF sprouting is seen with target granule cells at aberrant synapses recruiting *de novo* GluR2/GluR5 heteromeric KARs. Finally, in human epilepsy and animal models, astrocytic expression of GluK1, 2, 4, and 5 is reported.

## Introduction

The analog of glutamate *k*ainic *a*cid/kainate (KA) is a potent neurotoxin derived from the alga *Digenea simplex*. The word “Kainic” is derived from the Japanese “Kaininso” (“Makuri”), which means “the ghost of the sea”. KA was first isolated in 1953, from *D. simplex*, and therapeutically utilized, given its toxicity towards intestinal worms. Thereafter, KA was used to induce behavioral and electrophysiological seizures, establishing a model of temporal lobe epilepsy (TLE), where seizures originated in the hippocampal CA3 region (Nadler, 1981). Indeed, the toxin produces tissue damage and lesions that are reminiscent of those observed in human patients with TLE. However, while the acute effect of KA in producing seizures is well-established, its cogency in the clinically relevant chronic phase of TLE remains to be determined.

Kainate actions are largely mediated by the activation of kainate receptors (KARs) at which KA is a high affinity agonist. KARs, together with AMPA and NMDA receptors, comprise the ionotropic receptors family of glutamate receptors. Additionally, there is now considerable evidence that KARs have metabotropic/non-canonical actions (Rodríguez-Moreno and Sihra, 2007a,b). Given that metabotropic actions have also been described for AMPA, and recently for NMDA receptors (Valbuena and Lerma, 2016; Bouvier et al., 2018), the distinction between the signaling mediated by ionotropic and metabotropic glutamate receptors is increasingly blurred. KARs are specifically comprised of GluK1-GluK5 subunits. In expression systems, GluK1, GluK2, and GluK3 may form homomeric receptors, while GluK4 and GluK5 form heteromeric receptors in conjunction with GluK1–3 subunits. Native KARs are widely distributed in the brain (Bahn et al., 1994), with high densities of receptors found in the hippocampus, a key structure featuring in TLE. Hippocampal KARs containing GluK1 subunits are mostly expressed in interneurons, where the reduction of GABA release results in the increased excitability of glutamatergic principal neurons (Clarke et al., 1997; Rodríguez-Moreno et al., 1997, 2000; Rodríguez-Moreno and Lerma, 1998), or enhance inhibition by increasing GABA release (Cossart et al., 1998; Frerking et al., 1998; Jiang et al., 2001). GluK2 subunit containing KARs are mostly located in excitatory neurons, where they modulate glutamate release presynaptically (Chittajallu et al., 1996; Vignes et al., 1998; Contractor et al., 2000, 2001, 2003, Kamiya and Ozawa, 2000; Schmitz et al., 2000, 2001; Lauri et al., 2001; Rodríguez-Moreno and Sihra, 2004, 2013; Negrete-Díaz et al., 2006, 2007, 2012, Pinheiro et al., 2007; Scott et al., 2008; Fernandes et al., 2009; Lyon et al., 2011; Andrade-Talavera et al., 2012, 2013; reviewed in Sihra and Rodríguez-Moreno, 2013), and post-synaptically, underpin part of the synaptic component of excitatory transmission (Castillo et al., 1997; Vignes et al., 1997). This differential subcellular localization allows KARs to regulate the actions of neural circuits in the hippocampus.

Epilepsy is a chronic neurological disorder characterized by the occurrence of spontaneous recurrent seizures (prolonged and synchronized neuronal discharges). TLE is the most common form of human epilepsy. Recurrent seizures originate from diverse structures, usually the hippocampus, and sometimes may propagate to the amygdala and the entorhinal cortex (Lothman et al., 1991). Current antiepileptic drugs that are effective in controlling seizures in patients with TLE, either act on voltage- gated Na^+^ channels and Ca^2+^ channels, to modify neuronal excitability, or enhance GABAergic inhibition. However, although this anticonvulsant drug therapy reduces overall neuronal excitability in the brain, there are unwanted side effects (Perucca and Meador, 2005; Goldenberg, 2010). And, indeed, in many cases where TLE is severe, it is intractable to current drug therapy. Looking to alternative antiepileptic drug targets, while the GluK1 subunit-containing KAR represents a potential candidate (Rogawski et al., 2003), there is no anticonvulsant therapy based on KARs signaling that is currently in use in humans.

Models for seizures involving KAR activation can be acute or chronic. KA or pilocarpine injections represent acute models of epileptiform seizures. In chronic models, systemic administration of the aforementioned agents is continued for 1–3 days. In a high proportion of injected animals treated in this way, after a lag of some days or weeks without any seizures, a chronic phase of spontaneous recurrent limbic seizures is precipitated. These seizures increase in frequency and do not resolve (Ben-Ari, 1985; Leite et al., 1990). KA-induced seizures in animals produce patterns of activity-induced neuronal and cell loss, astrogliosis and hippocampal sclerosis of CA1 and CA3 pyramidal cells (PyC), that resemble characteristics of human TLE (Nadler et al., 1978; Ben-Ari, 1985).

## Kar Role in Acute Seizures

**FIGURE 1:**
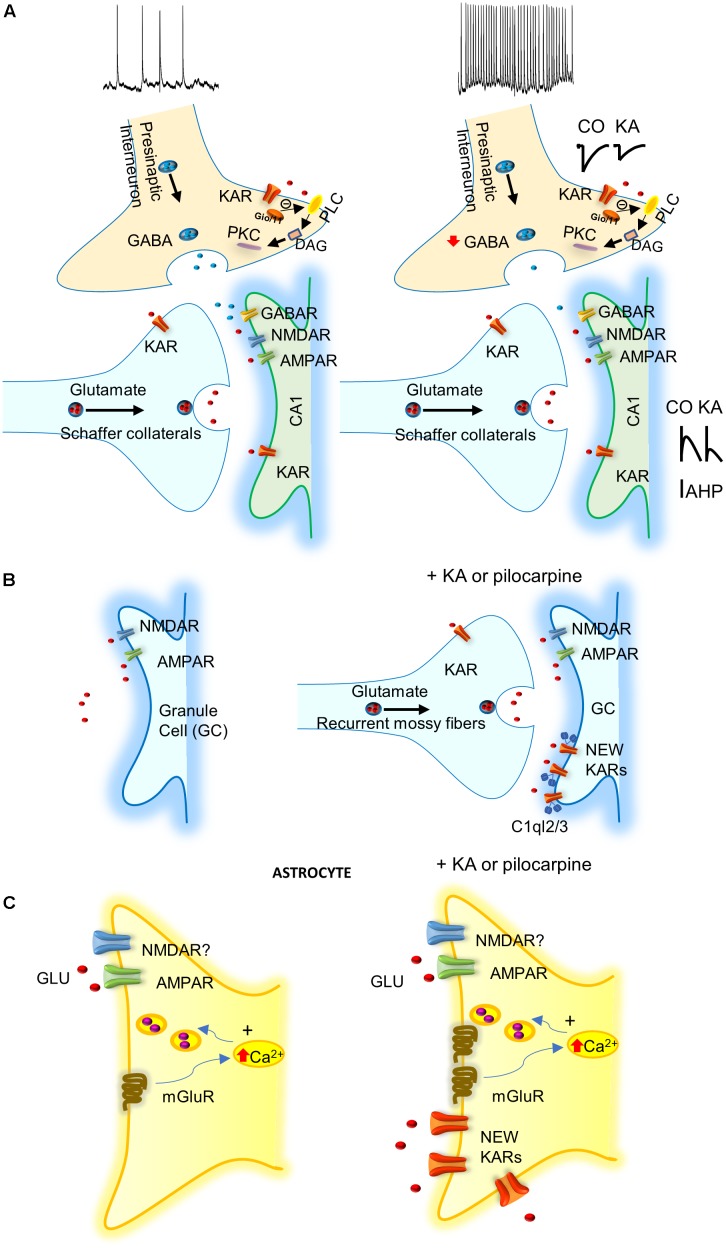
Possible mechanisms involving KARs in epilepsy. **(A)** KARs activation depress GABA release what together with the activation of post-synaptic KARs increase excitability of principal cells. IAHP, afterhyperpolarization potassium current is decreased by KARs activation increasing neuronal excitability. **(B)** KARs induce “reactive plasticity” and mediate the insertion of new post-synaptic KARs at granule cells (GCs) of dentate gyrus altering spiking. C1ql2/3 (C1q-like proteins, related to the C1q complement component) recruit KARs at GCs after pilocarpine treatment. **(C)** KA or pilocarpine treatments mediate the insertion of KARs in astrocytes that probably releases gliotransmitter’s to participate in protection of induction of epilepsy.

The majority of studies of KARs related to epilepsy have investigated the *acute* effects of KA-induced seizures (Rodríguez-Moreno et al., 1997; Mulle et al., 1998; Smolders et al., 2002; Fritsch et al., 2014). KA injection produces an acute epileptogenesis mediated by KAR-mediated suppression of presynaptic GABA release, together with post-synaptic KAR activation of glutamatergic neurons (Rodríguez-Moreno et al., 1997; reviewed in Lerma and Marques, 2013). However, to date, there is no explanation for the chronic effect of KA that continues some months after the KA treatment. Hippocampal interneurons in the CA1 region of the hippocampus possess GluK1 subunit containing KARs in the axonal compartment, as well as in the somatodendritic compartment (Paternain et al., 2000; Rodríguez-Moreno et al., 2000). KA at interneuron-interneuron synapses facilitates GABA release, and thus inhibitory drive (Cossart et al., 1998). At interneurons-principal cells synapses, hippocampal interneurons manifest a biphasic effect of KA. Activation of KARs by “high” doses of KA suppresses GABA release (Clarke et al., 1997; Rodríguez-Moreno et al., 1997), while stimulation of KARs by “low” KA concentrations, or ATPA (an agonist of GluK5 subunit containing KARs), facilitates GABA release (Jiang et al., 2001; Khalilov et al., 2002). However, contrary to what would be predicted by the latter observation, *in vivo*, the systemic administration of ATPA actually induced seizures in the hippocampus and the amygdala. This effect was contingent on the presence of GluK1 given that it was abolished in GluK1^-/-^ mice (Fritsch et al., 2014). Consistent with this, *in vivo* antagonism of KARs containing the GluK1 subunit, blocked seizures induced by the muscarinic receptor agonist pilocarpine (Smolders et al., 2002). These observations indicate that the regulation of inhibition is key for the potential action of KA on KARs and epilepsy. Thus, *in vivo*, the net effect of the activation of KARs seems to be an overall depression of GABA release leading to increased excitation and thereby epileptiform activity. This is, to date, one of the best pathophysiological demonstrations of the net effects of KA in epileptgenesis (Rodríguez-Moreno et al., 1997; **Figure 1A**). Post-synaptic KARs have also been found in the principal cells of the hippocampus. Here the KARs contain at least one GluK2 subunit, and their activation mediates the regulation of afterhyperpolarization. Alteration of the latter affects neuronal excitability and may therefore contribute to susceptibility to the pro-convulsant effects of KA (Melyan et al., 2002, 2004).

In agreement with a role of KARs containing GluK1 subunit in seizure induction, in a study of members of a family affected by idiopathic juvenile absence epilepsy, there were increased levels of Grik1 polymorphisms (Sander et al., 1997). Additionally data from TLE patients also showed that the GluK1 subunit is upregulated (Li et al., 2010), indicating that there is KAR reorganization occurring in human epilepsy. Mitigating against this, the AMPA/KAR antagonist NS1209, has been shown, in clinical Phase II studies, to alleviate refractory status epilepticus (Swanson, 2009).

In summary, in *ex vivo* and in *in vivo* models, in the hippocampus and the amygdala, KA has been found to reduce GABA release. This attenuates the inhibition of hippocampal pyramidal cells and is thereby posited to provoke epileptic activity. It has also been shown that the use of antagonists of GluK1 subunit-containing KARs prevented epileptic activity, thus corroborating a key role for synaptic inhibition in the KAR-induced seizures. Post-synaptic KARs activation mediate an increase in excitability. As a general mechanism then, the proposal is that the activation of KARs orchestrates an imbalance between excitation and inhibition, and this represent one way that KA activating KARs induces acute seizures (**Figure 1A**).

## Kars Role in Chronic Seizures

Since the 1970s, it has been known that the CA3 region of the hippocampus is a key area related to the origin of seizures (reviewed in Ben-Ari and Cossart, 2000). KARs containing the GluK2 subunit have been linked to limbic epilepsies because of its specific distribution in CA3 pyramidal neurons (Werner et al., 1991). Consistent with a direct role of KARs in the induction of epilepsy in this region, the ablation of GluK2 subunits in knockout studies reduced the sensitivity of the mice to develop seizures after KA injection (Mulle et al., 1998).

In animal models of TLE and human patients, neuronal tissue undergoes major reorganization, where some neurons die and others sprout and make aberrant connections (Ben-Ari et al., 2008). This “reactive plasticity” is well-described in the dentate gyrus of the hippocampus. Here, mossy fibers (MFs), the axons of granule cells (GCs) from the dentate gyrus and where KARs are highly expressed, undergo sprouting after KA injections and form a functional recurrent MF network (Tauck and Nadler, 1985; Represa et al., 1987; Sutula and Dudek, 2007). MF sprouting is therefore considered as one of the pathological characteristics of TLE in humans and animal models. Interestingly, at these aberrant synapses developed under pathological conditions, ‘*de novo*’ KARs are expressed in the GCs of the dentate gyrus, and these likely participate in the pathogenesis of TLE (Epsztein et al., 2005; Artinian et al., 2011, 2015). KA mediates glutamatergic currents at MF-GC synapses, and some half of the glutamatergic transmission has been proposed to be due to the insertion of these new KARs (Epsztein et al., 2005; Peret et al., 2014; reviewed in Vincent and Mulle, 2009; Crépel and Mulle, 2015). The consequence of the insertion of KARs in GC would be the generation of a hyperexcitable circuit in the hippocampal dentate gyrus. This may ultimately produce epileptiform activity, particularly when inhibition by GABAergic transmission is simultaneously depressed (Epsztein et al., 2005).

The aforementioned aberrantly inserted post-synaptic KARs are likely heteromeric receptors containing GluK2/GluK5 subunits, that can be blocked by the specific antagonist for post-synaptic MF KARs, UBP310 (Pinheiro et al., 2013). GluK2, 4, and 5 subunits have been found at post-synaptic sites (Mulle et al., 1998; Contractor et al., 2003; Fernandes et al., 2009), and are involved in generation of recurrent seizures in chronic epilepsy. Presynaptic MF KARs comprise GluK2 and GluK3 subunits, and mediate facilitation or depression of glutamate release, and are thereby involved in presynaptic plasticity (reviewed in Sihra and Rodríguez-Moreno, 2013; Sihra et al., 2014). The *de novo* KARs present in dentate gyrus GC impair temporal precision of EPSP-spike coupling and thus induce seizures by altering the basally sparse firing rate of dentate gyrus GCs (Artinian et al., 2011, 2015). Additionally it is known that the CA3 synaptic silencing attenuated KA induced seizures and network oscillations in the hippocampus (Yu et al., 2016).

The study of the role of the aforementioned new/aberrant KARs in the chronic phase of TLE in the pilocarpine model of chronic TLE showed that the inter-ictal and ictal events were reduced in mice lacking GluK2 subunit, but not those lacking GluK1, or with the use of a GluK2/GluK5 antagonist (Peret et al., 2014). These results suggest that these KARs may represent targets for antiepileptic drugs (**Figure 1B**). KARs also modulate intrinsic conductance by a metabotropic action at MF-CA3 synapses (Fisahn et al., 2004). It remains to be elucidated how aberrant KARs are recruited to the membrane and whether these abnormally expressed post-synaptic KARs in dentate gyrus GCs are activated by endogenous glutamate, to develop and propagate seizures in the hippocampus. However, it has recently been shown that C1q-like proteins (related to the C1q complement component) may have a role at MFs (Matsuda et al., 2016). C1ql2 and C1ql3 proteins produced by MFs have been demonstrated to serve as extracellular organizers to recruit functional post-synaptic KAR complexes to the CA3 pyramidal neurons and GCs (**Figure 1B**).

## Possible Role of Astrocytes in Ka Induced Seizures

In the recent years, evidence has accumulated to suggest that astrocytes may have an important role in KA-induced seizures. In tissue from patients with refractory TLE, an increase of KARs subunits GluK4 and GluK5 have been reported (Das et al., 2012). Surprisingly, KARs subunits GluK1, 2, 4, and 5 are all expressed in astrocytes, 1 week after the induction of status epilepticus in the CA1 region of the hippocampus of treated animals, but not in naïve animals. Further, GluK1 and GluK5 subunits remain enhanced in the chronic phase of epilepsy when spontaneous seizures occurs (Vargas et al., 2013). The role of the newly expressed KARs in the astrocytes is currently unclear. Indeed, it is unknown whether this recruitment of KARs in astrocytes impinges on the induction and propagation of seizures (as activation of these KARs might mediate the release of glutamate from astrocytes), or have just the opposite effect, viz. a protective role. Future work will be needed to determine the exact role of astrocytes and astrocytic KARs in epilepsy (**Figure 1C**).

## Future Directions

One important aspect that awaits resolution is whether the KARs involved in the induction of acute or chronic seizures are activated directly by endogenously released glutamate. Further, the precise mechanism by which the loss of excitatory/inhibitory balance induces seizures and their propagation requires evaluation. Importantly, the role of “reactive plasticity” in epilepsy, as a general or basic mechanism for epilepsy, needs to be expounded and clarified. Finally, the function of astrocytic KARs and astrocytes in epilepsy requires explication, as to whether their involvement is pro- or anti-epileptogenic.

In conclusion, three approximations are promising mechanisms in determining the role of KARs in epilepsy: (i) depression of inhibitory synaptic transmission and increase in excitatory transmission (i.e., disruption of excitation/inhibition balance); (ii) induction of reactive plasticity with new KARs changing network properties; and (iii) the emerging role of astrocytes in epilepsy. These three working hypotheses await further development to understand the exact role of the intriguing KARs in epilepsy etiology, and facilitate the elaboration of novel, and hopefully more efficacious, therapies for epilepsy.

## Author Contributions

All authors listed have made a substantial, direct and intellectual contribution to the work, and approved it for publication.

## Conflict of Interest Statement

The authors declare that the research was conducted in the absence of any commercial or financial relationships that could be construed as a potential conflict of interest.
